# The potential impact of ChatGPT in clinical and translational medicine

**DOI:** 10.1002/ctm2.1206

**Published:** 2023-02-28

**Authors:** Christian Baumgartner

**Affiliations:** ^1^ Graz University of Technology Institute of Health Care Engineering with European Testing Center of Medical Devices Graz Austria

1

‘The Ultimate Computer’, an episode of the legendary 1968 series Star Trek: The Original Series, in retrospect, put the spotlight on the unprecedented development of artificial intelligence (AI), with its enormous possibilities, but also limitations and dangers, without knowledge of the modern computational methods of the 21st century. I recently watched this episode under this premise and was stunned by the conversations about the benefits and dangers of artificial intelligence that were already taking place at that point.

In 2011, this episode was named by Forbes as one of the 10 best episodes of the series, dealing with the effects of advanced technology and, in particular, the dangers of artificial intelligence.^1^ Forbes commented: ‘*The Ultimate Computer’ (TOS) ‐ This is a pretty fascinating episode, especially considering the time period. Pre‐dating HAL by a few years, this episode features M‐5, a computer capable of learning and adapting that is given command of the Enterprise on a test run. Naturally, things don't go well, but it's an interesting exploration of the difference between human and machine intelligence. It also serves as a metaphor for people who lose their jobs due to mechanization’*.

Almost 55 years after this episode, the OpenAI project was released in November 2022. The question of the potential impact of ChatGPT in clinical and translational medicine has already raised and will continue to raise many questions that can only be hinted at in this commentary. The easiest way to approach this question is to ask ChatGPT itself. Therefore, I entered the following query: ‘What are the benefits, challenges, pitfalls and future perspectives of ChatGPT in clinical and translational medicine? Write a short assessment on this topic.’ ChatGPT has given the following answer:

*“ChatGPT, a state‐of‐the‐art language model developed by OpenAI, has the potential to revolutionize the way medical information is disseminated and processed in the clinical and translational medicine fields. Here are some of the key benefits, challenges, pitfalls and future perspectives of using ChatGPT in this context”*:

*Benefits*:

1. *Access to up‐to‐date information: ChatGPT can access vast amounts of medical data and provide real‐time, accurate answers to clinical questions*.
2. *Improved patient engagement: ChatGPT can provide patients with a convenient and accessible way to get answers to their medical questions and to track their health information*.^1^
3. *Reduced workload for healthcare providers: ChatGPT can help reduce the administrative burden on healthcare providers, allowing them to focus more on patient care*.

*Challenges*:

1. *Data privacy concerns: ChatGPT may raise privacy concerns as it has access to vast amounts of personal health information*.
2. *Inconsistent accuracy: ChatGPT may not always provide accurate answers, particularly for complex medical questions*.
3. *Bias in the training data: ChatGPT is only as good as the data it was trained on. If the training data are biased, the model may perpetuate that bias*.

*Pitfalls*:

1. *Misleading information: ChatGPT is not a substitute for a healthcare provider, and incorrect or misleading information may cause harm to patients*.
2. *Dependence on technology: ChatGPT may become a crutch for healthcare providers, reducing their ability to diagnose and treat patients without relying on technology*.

*Future perspectives*:

1. *Improved accuracy: As more data are collected and analyzed, ChatGPT's accuracy is likely to improve*.
2. *Integration with electronic health records (EHRs): ChatGPT has the potential to be integrated with EHRs, allowing for a more seamless exchange of information between healthcare providers and patients*.
3. *Personalized medicine: ChatGPT has the potential to provide personalized medical advice based on individual patient data and health histories*.
*In conclusion, ChatGPT has the potential to significantly impact the clinical and translational medicine fields by providing access to up‐to‐date information, improving patient engagement and reducing workloads for healthcare providers. However, there are also challenges and pitfalls to be considered and addressed, and continued research and development are needed to ensure that ChatGPT is used safely and effectively’*.

When repeating the same query (“Regenerate Response”), it is interesting to note that the structure of the text remains unchanged, but with some deviations and variations from the previous result (see Figure 1). These differences can be explained by the underlying structure and learning architecture as well as the parameter settings of the tool, making it difficult to reproduce repeated queries. One should know that ChatGPT is deep learning model that uses a transformer‐based neural network to generate human‐like text.

**FIGURE 1:**
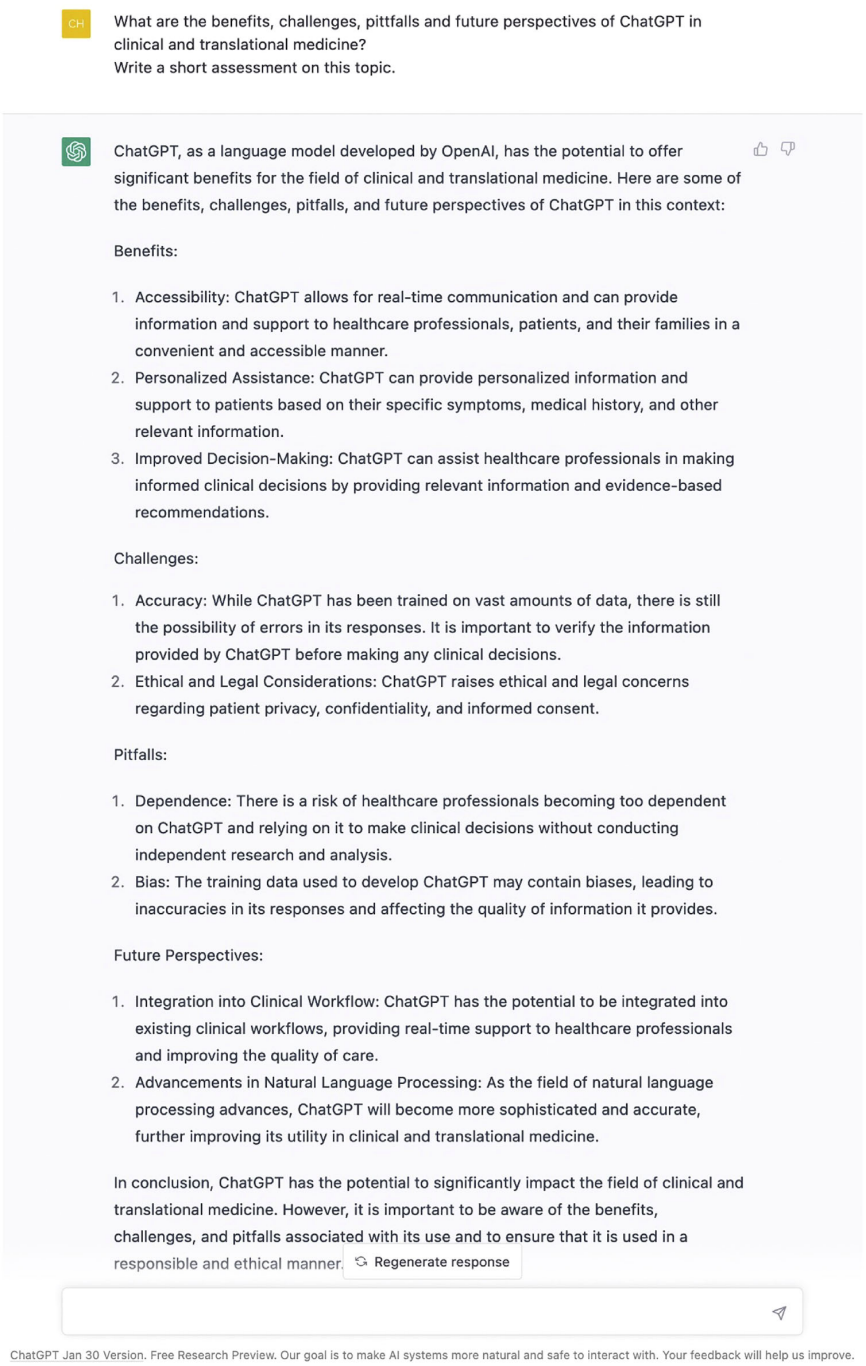
‘Regenerate Response’ to the same query. It shows some deviations from the previous result.

It is intriguing that the original question about the potential impact of ChatGPT in clinical and translational medicine seems to be almost answered by ChatGPT itself, yet there are some questions that need to be addressed here as a result of the global discussion that has begun about ChatGPT and its impact in healthcare. There is no doubt that the benefits and advantages of AI in medicine are beyond question, and have already made their way into everything from research to clinical applications. AI‐based algorithms in all areas from data‐driven preclinical research to medical decision support in the daily clinical practice, for example, through AI‐based interpretation of medical images for diagnosis and patient management, pattern recognition and pre‐interpretation of physiological and biophysical signals such as ECG, EEG, EMG and others, are already parts of standardized medical procedures according to GCP.

However, the challenges and pitfalls indicated by ChatGPT naturally raise many questions, including relevant medical ethics issues. Data privacy and security, incorrect or misleading information that may cause harm to patients. Otherwise, patients are well prepared for the doctor's interview and appear confident on relevant clinical information such as disease‐specific symptoms, medical history, and so on. However, this is often not in the interest of the attending physician and can lead to misunderstandings. In addition, the inconsistent accuracy of ChatGPT responses can lead to problems in education if ChatGPT is used without authorization. Some universities have already banned this tool. Medical staff in training and students could be influenced accordingly by ChatGPT and misinterpret medical knowledge or even make clinical decisions without independent and critical evaluation and validation.

The most critical technical issue is training data bias, as ChatGPT's underlying algorithms are only as good as the data they are trained on, and are therefore prone to errors in their responses. Consequently, it would be part of GCP to train and validate the ChatGPT algorithm, for example, for diagnosis and therapy‐accompanying applications on relevant, evidence‐based knowledge bases, before it can be used. Here, approaches and concepts as known from medical device approval should be considered, including requirements and usability engineering, risk assessment and clinical evaluation.

In summary, there are many other aspects that could be discussed in this commentary to illustrate the tremendous benefits of open AI, while not ignoring the consequences and implications of using ChatGPT unwisely. Returning to the introductory example of the science fiction episode ‘The Ultimate Computer’, the advantages and pitfalls of an AI‐controlled world were impressively portrayed in this story and have also proven to be real to this day. With the launch of the OpenAI project in 2022, the world has finally been catapulted into a new ‘age of artificial intelligence’, and humans are being called upon to take ultimate responsibility for these new technologies.

Once again, I would like to repeat the conclusion of ChatGPT:

*‘ChatGPT has the potential to significantly impact the clinical and translational medicine fields by providing access to up‐to‐date information, improving patient engagement and reducing workloads for healthcare providers. However, there are also challenges and pitfalls to be considered and addressed, and continued research and development are needed to ensure that ChatGPT is used safely and effectively’*.

In the hope and ethical obligation to improve the quality of life of patients.

## CONFLICT OF INTEREST STATEMENT

The authors declare no conflicts of interest.

## Footnotes

1
https://www.forbes.com/sites/alexknapp/2011/04/08/the‐10‐best‐singularity‐themed‐star‐trek‐episodes/?sh=4b9f250c340f

